# A Pan-Cyclophilin Inhibitor, CRV431, Decreases Fibrosis and Tumor Development in Chronic Liver Disease Models[Fn FN1]

**DOI:** 10.1124/jpet.119.261099

**Published:** 2019-11

**Authors:** Joseph Kuo, Michael Bobardt, Udayan Chatterji, Patrick R. Mayo, Daniel J. Trepanier, Robert T. Foster, Philippe Gallay, Daren R. Ure

**Affiliations:** Department of Immunology and Microbiology, Scripps Research Institute, La Jolla, California (J.K., M.B., U.C., P.G.); and Hepion Pharmaceuticals, Edison, New Jersey (P.R.M., D.J.T., R.T.F., D.R.U.)

## Abstract

Previous studies show that cyclophilins contribute to many pathologic processes, and cyclophilin inhibitors demonstrate therapeutic activities in many experimental models. However, no drug with cyclophilin inhibition as the primary mode of action has advanced completely through clinical development to market. In this study, we present findings on the cyclophilin inhibitor, CRV431, that highlight its potential as a drug candidate for chronic liver diseases. CRV431 was found to potently inhibit all cyclophilin isoforms tested—A, B, D, and G. Inhibitory constant or IC_50_ values ranged from 1 to 7 nM, which was up to 13 times more potent than the parent compound, cyclosporine A (CsA), from which CRV431 was derived. Other CRV431 advantages over CsA as a nontransplant drug candidate were significantly diminished immunosuppressive activity, less drug transporter inhibition, and reduced cytotoxicity potential. Oral dosing to mice and rats led to good blood exposures and a 5- to 15-fold accumulation of CRV431 in liver compared with blood concentrations across a wide range of CRV431 dosing levels. Most importantly, CRV431 decreased liver fibrosis in a 6-week carbon tetrachloride model and in a mouse model of nonalcoholic steatohepatitis (NASH). Additionally, CRV431 administration during a late, oncogenic stage of the NASH disease model resulted in a 50% reduction in the number and size of liver tumors. These findings are consistent with CRV431 targeting fibrosis and cancer through multiple, cyclophilin-mediated mechanisms and support the development of CRV431 as a safe and effective drug candidate for liver diseases.

**SIGNIFICANCE STATEMENT:**

Cyclophilin inhibitors have demonstrated therapeutic activities in many disease models, but no drug candidates have yet advanced completely through development to market. In this study, CRV431 is shown to potently inhibit multiple cyclophilin isoforms, possess several optimized pharmacological properties, and decrease liver fibrosis and tumors in mouse models of chronic liver disease, which highlights its potential to be the first approved drug primarily targeting cyclophilin isomerases.

## Introduction

Cyclophilin A (Cyp A) was first isolated in 1984 and fittingly named for its feature characteristic—binding to the potent immunosuppressant, cyclosporin A (CsA). Cyp A is also known as peptidyl prolyl isomerase A (PPIA) because its primary biochemical activity is catalytic regulation of *cis-trans* isomerization of X-proline peptide bonds (where X represents any amino acid), which are important for protein folding and function. Eighteen human proteins with cyclophilin isomerase domains exist and occupy many cellular compartments (Davis et al., 2010; Lavin and McGee, 2015). The best described isoforms include Cyp A (PPIA; cytosol), cyclophilin B (Cyp B; peptidyl prolyl isomerase B; endoplasmic reticulum), and cyclophilin D (Cyp D; peptidyl prolyl isomerase F; mitochondria). Cyclophilins have important roles in normal physiologic function, but they also participate in many pathologic processes (Nigro et al., 2013; Naoumov, 2014; Xue et al., 2018; Briston et al., 2019). For example, Cyp D is a primary inducer of mitochondrial permeability transition that leads to cell death after a variety of cellular insults. Cyp A has been evolutionarily recruited into the life cycles of many viruses such as hepatitis B and C viruses (Dawar et al., 2017a). Overexpression of cyclophilins has been observed in many types of cancer, which appears to facilitate adaptation to hypoxia and elevated anabolic demands (Lavin and McGee, 2015). Extracellular Cyp A released from injured or dying cells can be proinflammatory through its binding to CD147. Cyp B, although important for collagen production and maturation throughout development, may exacerbate fibrotic pathologies characterized by excessive collagen production. Thus, pharmacological inhibitors of cyclophilins have the potential to be broadly therapeutic across a spectrum of diseases and disorders.

Two major pathologies to which cyclophilins are believed to contribute are fibrosis and cancer. In the liver, fibrosis commonly develops in all the major forms of chronic hepatitis—alcoholic, nonalcoholic, and viral—and is a primary predictor of cirrhosis, hepatocellular carcinoma (HCC), and mortality. Excessive deposition of extracellular matrix can profoundly change the anatomy and physiology of the liver and create an environment that promotes malignancy. HCC is the most common type of primary liver cancer, has a poor prognosis, and annually accounts for approximately 800,000 deaths worldwide (Kulik and El-Serag, 2019). New treatments that positively shift the fibrogenesis–fibrolysis dynamic toward decreasing fibrosis and lowering the risk of HCC are urgently needed.

The most thoroughly characterized chemical class of cyclophilin inhibitors are the cyclosporins. The prototypical inhibitor, CsA, is an 11-amino-acid cyclic peptide that revolutionized solid organ transplantation after its approval as an immunosuppressant in 1983. The mechanism of immunosuppression is binding of CsA to Cyp A, followed by CsA–Cyp A dimer binding to, and inhibition of the lymphocyte-activating phosphatase, calcineurin. Although CsA is a potent inhibitor of cyclophilins, its immunosuppressive activity largely limits its therapeutic use as a cyclophilin inhibitor. To address this limitation, many compounds have been produced that antagonize cyclophilins, but without significant calcineurin inhibition (Sweeney et al., 2014; Dunyak and Gestwicki, 2016). Nonimmunosuppressive analogs of CsA comprise the largest class, and notable representatives are valspodar, NIM811, EDP-546, SCY635, MM284, and alisporivir (DEBIO-025). Alisporivir demonstrated the most clinical potential by advancing through Phase 2 clinical trials with robust antiviral activity toward hepatitis C virus (Buti et al., 2015; Pawlotsky et al., 2015). Cyclophilin inhibitors also have been derived from other chemical platforms—small molecules or derivatives of the macrolide, sanglifehrin A—but they often have shown lower potency than cyclosporin compounds, poor bioavailability, or have not been extensively characterized (Moss et al., 2012; Sweeney et al., 2014; Yan et al., 2015). Despite this diversity of cyclophilin inhibitors, none have advanced completely through clinical development to market.

CRV431 is a CsA analog that is unique from most previously described derivatives, as a result of chemical substitutions made at amino acids 1 and 3 of the cyclosporine ring (Trepanier et al., 2018). Its antiviral activities toward hepatitis B virus, hepatitis C virus, and human immunodeficiency virus-1 and other properties have previously been reported (Gallay et al., 2015, 2019). The present report further documents activities of CRV431 that distinguish it from other members of the cyclosporin class. The data reinforce the potential of pan-cyclophilin inhibitors as safe, therapeutic agents. We show that CRV431 decreases liver fibrosis in two animal models and decreases liver tumor burden in a mouse model of nonalcoholic steatohepatitis (NASH), which highlights its potential as a treatment of liver disease of various etiologies.

## Materials and Methods

### 

#### CRV431 and CsA Solutions.

For all in vitro experiments, CRV431 and CsA stock solutions in DMSO (D2650; Sigma-Aldrich) were prepared first at concentrations up to 80 mM, followed by at least 500-fold dilution into buffers or cell culture medium to create the working solutions. DMSO was used as the vehicle control in all in vitro experiments at the same dilutions as were used in making the CRV431 working solutions. The maximum working solution concentration of CRV431 in aqueous medium was 80 *µ*M (cytotoxicity assays), which was determined by high performance liquid chromatography analysis to be below the limit of aqueous solubility (115 *µ*M) and completely soluble (Trepanier et al., 2018). The maximum attempted working solution concentration of CsA in aqueous medium was 80 *µ*M (cytotoxicity assays), but high performance liquid chromatography analysis determined a maximum CsA solubility of 31.9 *µ*M (Trepanier et al., 2018).

#### Cyclophilin Isomerase Assay.

Cyclophilin isomerase activity was measured with an assay described by Kofron et al. (1991) with adaptations to 96-well plates. In this assay cyclophilins catalyze *cis*-to-*trans* isomerization of a proline peptide bond in a nitroanalide-coupled peptide substrate followed by cleavage of the *trans*-conformer peptide by *α*-chymotrypsin, release of the *p*-nitroaniline chromogen, and quantitation of *p*-nitroaniline by absorbance at 410 nm. All solutions were pre-equilibrated, and all assays were conducted at 4°C. Plates were preloaded with 5 *µ*l peptide solution consisting of N-succinyl-alanine-alanine-proline-phenylalanine-*p*-nitroanilide (S7388; Sigma-Aldrich) and 400 mM lithium chloride (310468; Sigma-Aldrich) in anhydrous trifluoroethanol (T63002; Sigma-Aldrich). Eight peptide concentrations spanning 100–1000 *µ*M were tested for inhibitory constant (Ki) determinations, and a single peptide concentration, 150 *µ*M, was used for IC_50_ determinations. *Cis*-peptide represented 55% of the peptide isomers, based on initial reaction bursts, and therefore peptide concentration was corrected to 55% of input in subsequent calculations. Reactions were initiated by thoroughly mixing in 95 *µ*l reaction mix consisting of 50 mM HEPES (pH 8.0); 100 mM NaCl; 20 nM recombinant Cyp A (3589-CAB; R&D Systems), Cyp B (CLENZ313; Cedarlane Laboratories), Cyp D (CLENZ385; Cedarlane Laboratories), or cyclophilin G (CLENZ463; Cedarlane Laboratories); 1 mg/ml *α*-chymotrypsin (C4129; Sigma-Aldrich); 5 mg/ml human serum albumin (A1653; Sigma-Aldrich); and 3-fold serial dilutions of CRV431 and CsA spanning 0.05–1000 nM. Absorbance measurements with a BMG Polarstar Galaxy plate reader were begun immediately and made at 6-second intervals for 6 minutes. Reaction curves were represented by increases in optical density 410 nm as a function of reaction time. For Ki determinations, initial reaction velocities were obtained from the reaction curves and plotted relative to substrate concentration, and the curves were fitted to the enzyme competitive inhibition model (GraphPad Prism) to derive Ki, maximum velocity (Vmax), and Michaelis constant values. For IC_50_ determinations, the reaction curves were fitted to one-phase exponential association regressions to obtain first-order rate constants. Catalytic rate constants were calculated by subtracting the uncatalyzed reaction rate constants (no cyclophilin plotted as a function of CRV431 or CsA concentration and fitted by sigmoidal dose–response regressions to obtain IC_50_ values).

#### Nuclear Factor of Activated T Cells and Interleukin-2 Luciferase Reporter Assays.

The luciferase reporter assays employed Jurkat cells stably transfected with gene constructs consisting of a luciferase gene plus human nuclear factor of activated T cells (NFAT) or interleukin-2 (IL-2) promoter elements (J1621 and J1651; Promega). The NFAT construct contained a DNA binding site only for NFAT, whereas the IL-2 construct contained binding sites for NFAT, nuclear factor *κ*-light-chain-enhancer of activated B cells, and activating protein-1 transcription factors. Cells were cultured in 96-well plates at 100,000 cells/well in RPMI 1640 medium (11875-093; Thermo Fisher) supplemented with 5% FBS (12483020; Thermo Fisher). Cells were activated either by precoating high protein-binding plates with 3 *µ*g/ml anti-CD3 (UCHT clone) and anti-CD28 (CD28.2 clone) (BioLegend LEAF antibodies) or by adding phorbol myristate acetate (PMA; P8139; Sigma-Aldrich) to 15 ng/ml and A23187 ionophore (C7522; Sigma-Aldrich) to 0.75 *µ*M. DMSO, CRV431, or CsA was included across a range of concentrations. Cells were harvested after 18 hours of incubation, and luciferase was quantified with Promega Bio-Glo reagent (G7940; Promega) and measurement with a luminescence plate reader. Data were normalized to DMSO controls (100% stimulation) and to wells without antibody or PMA plus A23187 stimulation.

#### Carboxyfluorescein Succinimidyl Ester–Peripheral Blood Mononuclear Cell Proliferation.

Peripheral blood mononuclear cells (PBMC) were isolated by Ficoll Paque Plus centrifugation (17144003; GE Life Sciences) of venous blood from a healthy human male volunteer. Cells were incubated for 5 minutes with 5 *µ*g/ml carboxyfluorescein diacetate succinimidyl ester (C1157; Thermo Fisher) to produce stable intracellular carboxyfluorescein succinimidyl ester (CFSE) labeling (CFSE-PBMC). CFSE-PBMC were incubated for 3 days in RPMI 1640 medium supplemented with 5% FBS, 100 U/ml penicillin, 100 *µ*g/ml streptomycin (15140122; Thermo Fisher), and drug treatments (CRV431, CsA, or DMSO vehicle) in 96-well high protein-binding plates (275,000 cells/well) precoated with 2 *µ*g/ml anti-CD3 antibody (UCHT clone) to cross-link lymphocyte CD3 T cell receptor and stimulate T cell division. Cells were collected after 3 days of culture incubation, and flow cytometry was used to measure the partitioning of CFSE into daughter cells arising from cell division (discrete cell populations with successively halved CFSE content). Daughter cells as a percentage of all CFSE-labeled cells were normalized to DMSO treatment (100% stimulation) and wells without CD3 antibody coating (nonstimulated).

#### CRV431 Pharmacokinetics and Distribution.

Male and female Sprague–Dawley rats and male C57BL/6 mice from the carbon tetrachloride and NASH studies were used to evaluate CRV431 pharmacokinetics and distribution. CRV431 and CsA were dissolved in self-microemulsifying drug vehicles (Trepanier et al., 2018) and administered by oral gavage. Terminal whole blood and livers were collected and frozen until drug extraction. Liver samples were homogenized in PBS or 1% formic acid (0.1 g–0.4 g liver sample per ml solvent) with bead-based homogenizers (TissueLyser or Beadruptor). CRV431 and CsA concentration standards for sample quantitation were made by spiking the compounds into blood and liver matrices. Sample and standard compounds were extracted from blood and liver homogenates using a zinc sulfate/methanol precipitation method and quantified by liquid chromatography–electrospray ionization–mass spectrometry, as previously described (Trepanier et al., 2018). Liver CRV431 concentrations were normalized to the masses of the liver samples.

#### Drug Transporter Inhibition.

CRV431 inhibition of drug transporters was assessed by measuring the transport and/or accumulation of transporter substrate probes in three types of test systems (Supplemental Table 1). Briefly, PGP and breast cancer resistance protein inhibition was assessed by measuring the bidirectional transport of substrates across cell monolayers in transwell culture plates. Efflux ratios were represented as (apical-to-basal flux)/(basal-to-apical flux), and efflux ratios were plotted relative to CRV431 concentration to derive IC_50_ values. Bile salt export pump (BSEP) and multidrug resistance protein (MRP)1 inhibition was assessed by measuring substrate accumulation in membrane vesicles prepared from insect Sf9 cells and recombinantly expressing BSEP and MRP2 (Genomembrane). Inhibition of all other transporters was assessed by measuring substrate accumulation in cells recombinantly expressing the transporter. Known inhibitors of each transporter were used as positive controls for the assays. CRV431 was tested at concentrations of 0.1, 0.3, 1, 3, 10, 30, and 50 *µ*M. Specific, ATP-dependent uptake was determined by subtracting uptake in negative control vesicles and cells not expressing the transporters, and secondly by subtracting uptake in the absence of ATP in the assays. Specific, ATP-dependent uptake was plotted as a function of CRV431 concentrations and fitted to nonlinear regressions to obtain IC_50_ values.

#### Cytotoxicity Analysis.

CRV431 cytotoxicity was evaluated in cell culture with six human cell types: Jurkat E6.1 lymphocyte cell line (88042803, male; Sigma-Aldrich), HepaRG hepatocyte cell line (HPRGC10, female; Life Technologies), primary renal epithelial cells (CC-2556, unknown gender; Lonza), primary dermal fibroblasts (CC-2511, female; Lonza), primary bronchial smooth muscle cells (CC-2576, male; Lonza), and primary umbilical vein endothelial cells (CC-2519, female; Lonza). Jurkat cells (suspension cell type) were plated at 35,000 cells/well in 96-well plates. All other cell types (adherent cell types) were plated 2500–10,000 cells/well (adherent cell types) in GelCol bovine collagen-coated (5167; Advanced Biomatrix) 96-well Nunc Edge 2.0 evaporation barrier plates (14387223; Thermo Scientific). Cell culture medium and culture conditions followed suppliers’ recommendations. Cultures were maintained for 3 days without medium change in the presence of DMSO drug vehicle, CsA, or CRV431 from 0.6 to 80 *µ*M. The cell cultures demonstrated less than 50% confluency at plating and 90%–100% confluency in DMSO control wells, indicating that cells were in a proliferation phase with little contact inhibition for most of the culture period. Cell viability was assessed at the end of the 3-day culture period using the In Vitro Toxicology Assay (Tox8-1KT; Sigma-Aldrich). In this assay the oxidoreduction indicator dye, resazurin, is colorometrically converted in proportion to the number of viable cells and measured by absorbance or fluorescence. Fluorescence was measured (excitation 540 nm; emission 590 nm) with a BMG Polarstar Galaxy Microplate Reader.

#### Mouse Studies.

Experimental protocols were approved by the Institutional Animal Care and Use Committee of Scripps Research Institute (La Jolla, CA) and adhered to guidelines from the National Institutes of Health (Bethesda, MD). Female pregnant E14 C57BL/6J mice were purchased from the Department of Animal Resource’s Rodent Breeding Colony (Scripps Research Institute), with pups used for experimentation. All mice were kept at the vivarium in Scripps Research Institute’s Department of Immunology & Microbiology (La Jolla, CA). Five mice were housed per cage with rolled newspaper as bedding and enriched with paper chunks for shredding and pultruded carbon tubes. Mice were maintained under a pathogen-free condition at 21°C with food and water provided ad libitum during daily cycles of 12 hours of light and darkness.

Carbon tetrachloride (CCl_4_)–induced liver fibrosis was conducted in C57BL/6J male mice starting at 7 weeks of age. Mice were injected intraperitoneally twice per week with 0.5–0.75 ml/kg CCL_4_ (289116; Sigma-Aldrich) and corn oil (0.05 ml total dose per mouse) for 6 weeks. CRV431, obeticholic acid (OCA, HY-12222; MedChemExpress), and vehicle were administered by daily oral gavage for the entire duration of the study. CRV431 and OCA were dissolved in a self-microemulsifying drug vehicle (Trepanier et al., 2018), and then diluted with PBS. CRV431 was administered at 50 mg/kg per day, and OCA was administered at 10 mg/kg per day.

NASH was initiated in 2-day-old C57BL/6J male mice by intraperitoneal injection with 200 *μ*g streptozotocin (S0130; Sigma-Aldrich) to disrupt pancreatic *β* cells, induce diabetes, and promote adiposity in the liver. Mice were weaned at 3 weeks of age and begun on a high-fat diet with 60%kcal fat (D12492N; Research Diets) for the duration of the studies. Additionally, mice with a regular diet (D12450KN; Research Diets) and without streptozotocin were included as negative controls. CRV431 was dissolved in a self-microemulsifying drug vehicle and then diluted with PBS and administered daily by oral gavage at 50 mg/kg per day. The start time and duration of vehicle and CRV431 treatments varied depending on the study.

Blood and livers were collected at the end of the NASH and CCl_4_ studies. In some studies, serum or plasma was isolated from freshly-collected blood, whereas in other studies blood was frozen for later analysis of CRV431 concentration. Livers were immersion-fixed in zinc-formalin and paraffin-embedded for histologic processing. Liver sections were stained with hematoxylin and eosin for assessment of inflammation, ballooning, and steatosis, and with sirius red for assessment of collagen I and III (fibrosis). Sirius red staining was quantified by blinded analysis in one section per liver using ImageJ software. Scoring of steatosis, ballooning, and inflammation (sum of portal and lobular inflammation) in liver sections was based on criteria and training by a board-certified pathologist. Table 1 shows criteria used for the histologic scoring.

**TABLE 1:** 

Score	Steatosis	Ballooning	Portal Inflammation	Lobular Inflammation
0	<5%	No ballooning	None/Minimal	No foci
1	5%–33%	Few balloon cells	Significant	<2 foci
2	33%–66%	Many/Prominent		2–4 foci
3	>66%			>4 foci

Liver tumors were quantified in freshly collected livers at sacrifice in one NASH study ending at 30 weeks of age. Liver tumors were counted and their diameters measured with a ruler and classified as small (>0.1 cm diameter, but not exceeding 0.5 cm), medium (0.5–1 cm diameter), or large (>1 cm diameter). Liver tumor burden scores (0–7 scale) were assigned to each liver based on criteria shown in Table 2.

**TABLE 2:** 

Score	Description
0	No detectable tumor nodules
1	One to four small nodules, and no medium or large nodules
2	More than four small nodules, and no medium or large nodules
3	Unlimited number of small nodules, plus one to two medium, and no large nodules
4	Unlimited number of small nodules, plus three or more medium, and no large nodules
5	Unlimited number of small and medium nodules, but no more than one large nodule
6	Unlimited number of small and medium nodules, but no more than two large nodules
7	Unlimited number of small and medium nodules, and three or more large nodules

## Results

### 

#### CRV431 Potently Inhibits Multiple Cyclophilin Isoforms.

Cyclophilin isomerase inhibition by CRV431 and CsA was assessed in a widely used enzymatic assay with recombinant cyclophilins (Kofron et al., 1991). CRV431 and CsA Ki toward cyclophilin A were determined by fitting substrate concentration-initial reaction velocity curves (multiple substrate concentrations) to the competitive enzyme inhibition model. These experiments yielded Ki values of 1.3 nM for CRV431 and 17.2 nM for CsA, indicating a 13-fold higher potency for CRV431. Maximum enzyme velocities (Vmax) in the presence of DMSO control, CRV431, or CsA were calculated to be 5.4, 5.3, and 4.7 *µ*mol/s per nanomole Cyp A, respectively, with a mean value of 5.1 *µ*mol/s per nanomole Cyp A. The similar Vmax values are consistent with the known competitive model of cyclophilin inhibition by cyclosporins (Fischer et al., 1989). In the absence of inhibitor, the Michaelis constant value was 0.94 *µ*M.

Next, we evaluated CRV431 inhibition of three additional cyclophilin isoforms—B, D, and G (Table 3). These assays were performed with only one peptide substrate concentration, 160 *µ*M, which therefore described CRV431 or CsA potencies as IC_50_ values. The measured IC_50_ values approximated the Ki values for Cyp A inhibition, indicating that IC_50_ values obtained in these assay conditions closely reflected the true inhibitory potencies of the compounds. Isomerase reactions performed with all the cyclophilin isoforms resulted in IC_50_ values of 2.5–7.3 nM for CRV431 and 10–28 nM for CsA (Table 3). Thus, cyclophilins A (PPIA), B (peptidyl prolyl isomerase B), D (peptidyl prolyl isomerase F), and G (peptidyl prolyl isomerase G) all were inhibited by CRV431 with low-nanomolar affinity, and CRV431 was 3.6–10.2 times more potent than CsA across all the isoforms.

**TABLE 3:** Inhibition of cyclophilin

Cyclophilin Isoform	CsA IC_50_ (nM)	CRV431 IC_50_ (nM)	IC_50_ Fold Difference
PPIA (Cyp A)	25.6	2.5	10.2
PPIB (Cyp B)	11.5	3.1	3.7
PPIF (Cyp D)	10.1	2.8	3.6
PPIG (Cyp G)	27.7	7.3	3.8

PPIB, peptidyl prolyl isomerase B; PPIF, peptidyl prolyl isomerase F; PPIG, peptidyl prolyl isomerase G.

#### CRV431 Demonstrates Low In Vitro Immunosuppressive Activity.

**Fig. 1.**
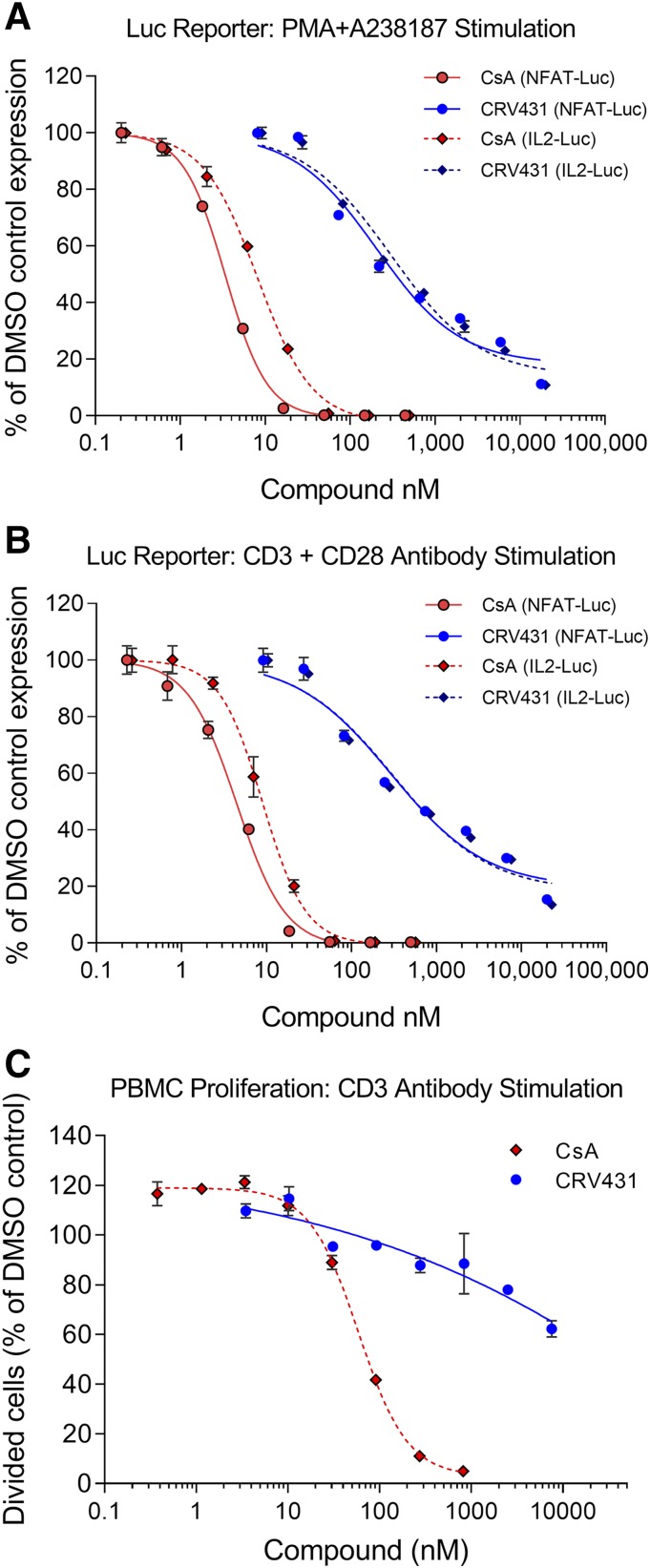
In vitro immunosuppression-related assays. (A and B) Jurkat T cells stably transfected with a luciferase reporter driven by NFAT or IL-2 promoter were stimulated with PMA and A23187 calcium ionophore (A) or immobilized CD3 and CD28 antibodies (B) in the presence of CRV431, CsA, or DMSO vehicle. Luciferase expression was measured after 18-hour stimulation. (C) Human peripheral blood mononuclear cells were labeled with CFSE and stimulated for 3 days by incubation with immobilized CD3 antibody in the presence of CRV431, CsA, or DMSO vehicle. The percentage of cells that underwent cell division after 3 days was determined by flow cytometric measurement of CFSE partitioning to daughter cells. Means and S.D. shown (*N* = 2 to 3 replicates per symbol).

Immunosuppression-related activity was assessed with two types of in vitro assays. The first type was Jurkat T lymphocytes stably transfected with luciferase reporter genes driven by one of two DNA promoter constructs. In one of the Jurkat lines, the promoter construct was the binding sequence for human nuclear factor of activated T cells (NFAT), a transcription factor that is a primary calcineurin target and mediator of lymphocyte activation. In the second Jurkat line, the promoter construct was the IL-2 promoter that contains binding sequences for NFAT and two other transcription factors, nuclear factor *κ*-light-chain-enhancer of activated B cells and activating protein-1. Both cell lines were stimulated for 18 hours in two ways: 1) addition of PMA plus A23817 calcium ionophore, and 2) incubation in culture plates precoated with antibodies to CD3 and CD28 to mimic physiologic lymphocyte stimulation. Luciferase production and inhibition by CRV431 and CsA were nearly identical for both types of stimulation (Fig. 1; Table 4). CsA was able to completely suppress Jurkat activation with IC_50_ values ranging from 3 to 9 nM. CRV431 showed a shallower concentration-dependence inhibition curve and when tested up to 20 *µ*M was not able to completely suppress activation. Nonlinear regression analysis gave CRV431 IC_50_ values ranging from 205 to 311 nM, but estimates of 50% inhibition derived directly from the graphs were 340–500 nM. Based on the ratios of CRV431 and CsA IC_50_, CRV431 was approximately 35 times less potent than CsA on Jurkat–IL-2 cells and 63 times less potent than CsA on Jurkat–NFAT cells, but these differences may be underestimates of CRV431’s lower potency in these cells.

**TABLE 4:** Drug potencies in immunosuppression-related luciferase reporter assays

Lymphocyte Stimulation	NFAT–Luc CsA IC_50_ (nM)	NFAT–Luc CRV431 IC_50_ (nM)	NFAT–Luc IC_50_ Fold Difference	IL-2–Luc CsA IC_50_ (nM)	IL-2–Luc CRV431 IC_50_ (nM)	IL-2–Luc IC_50_ Fold Difference
PMA + A23187	3.4	204.6	60.2	8.1	300.3	36.9
CD3 + CD28 antibodies	4.5	292.2	65.4	9.0	311.5	34.6

The second type of immunosuppression assay consisted of human PMBC that were stained with CFSE and cultured for 3 days with immobilized CD3 antibody to stimulate proliferation of the T cells. Cell proliferation was measured by flow cytometric quantitation of daughter cell populations with successively halved content of CFSE. CsA blocked lymphocyte proliferation with an IC_50_ of 59 nM, whereas CRV431 weakly inhibited proliferation. The highest tested CRV431 concentration of 10 *µ*M inhibited proliferation by only 40%, which negated IC_50_ determination. Also, the concentration–effect curve was very shallow, in similarity to the CRV431 effects in the Jurkat cell lines. Together, the data from these multiple assays indicate that CRV431 has significantly diminished immunosuppression potency, which does not exceed 3% of CsA levels (35-fold difference).

#### CRV431 Demonstrates Lower Cytotoxicity Potential Than CsA.

**Fig. 2.**
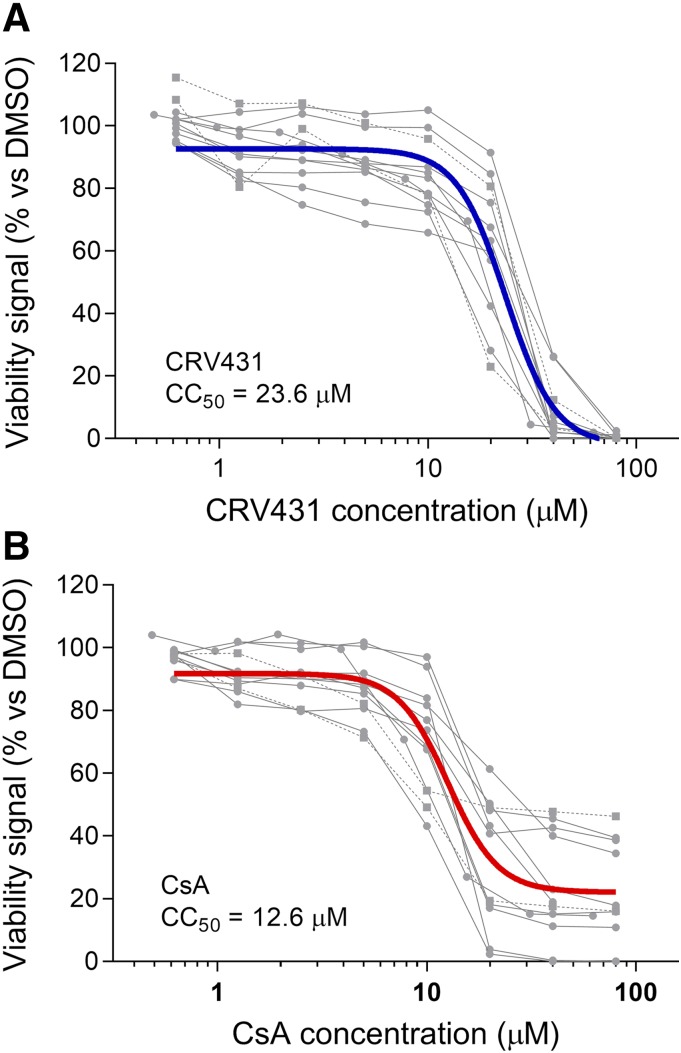
Cytotoxicity in human cells. Six human cell types were cultured for 3 days with CRV431 (A), CsA (B), or DMSO vehicle (A and B), followed by measurement of cell viability with a resazurin-based viability assay. Cytotoxicity curves for each independent experiment (gray symbols; one to two replicate experiments per cell line) and regression analyses of the means (black curves) are shown. The cell types included Jurkat lymphocyte cell line, HepaRG hepatocyte cell line, primary renal epithelial cells, primary dermal fibroblasts, primary bronchial smooth muscle cells, and primary umbilical vein endothelial cells.

CRV431 and CsA cytotoxicities were evaluated in six human cell types—Jurkat lymphocyte cell line, HepaRG hepatocyte cell line, primary renal epithelial cells, primary dermal fibroblasts, primary bronchial smooth muscle cells, and primary umbilical vein endothelial cells (Fig 2). Cell viability was measured after 3 days of culture and incubation with compounds up to 80 *µ*M. CRV431 caused 100% lethality at the highest concentrations tested, and the mean CC_50_ was 23.6 *µ*M. Maximum cytotoxicity of CsA was reached at 30 *µ*M but did not reach 100% lethality in most experiments because CsA reached its limit of aqueous solubility at approximately 32 *µ*M, as compared with CRV431’s aqueous solubility of 115 *µ*M (Trepanier et al., 2018). The mean CsA CC_50_ from all experiments was 12.6 *µ*M, indicating that CRV431 had lower cytotoxicity potential than CsA.

#### Improved Drug Transporter Inhibition Profile for CRV431.

Cyclosporins as a class are known to influence the pharmacokinetics of certain drugs and endogenous molecules by inhibiting their influx or efflux from cells through drug transporters. We therefore investigated inhibition of a panel of transporters by CRV431 in comparison with CsA. Three types of test systems were used—membrane-derived vesicles, conventional cell cultures, and transwell cultures (Table 5). Each test system expressed an individual transporter, and data were validated by comparison with vesicles and cells without the transfected transporter and by use of known transporter inhibitors as positive controls. Four transporters were not inhibited by CsA or CRV431 at the highest tested concentration of 50 *µ*M—OAT1, OCT2, MATE-1, and MATE-2. Only one transporter, OAT1, was inhibited by CRV431 more potently than CsA, with a 21-times lower IC_50_. CRV431 showed a small advantage over CsA toward BSEP and MRP2, demonstrating 2- to 3-fold higher IC_50_ values, and a more significant advantage toward BCRP, OATP1B1, and OATP1B3, with approximately 7-fold higher IC_50_ than CsA. The most significant advantage of CRV431 was toward NTCP and PGP , in which the CRV431 IC_50_ was 45 to 50 times higher than the CsA IC_50_. These data show that CRV431 is less inhibitory than CsA toward most membrane transporters and predict that CRV431 should have few drug–drug interactions and less impact on the transport of endogenous molecules such as bile salts and bilirubin.

**TABLE 5:** Drug transporter inhibition

Transporter	Test System	CRV431 IC_50_ (*µ*M)	CsA IC_50_ (*µ*M)	CRV431 Inhibitory Potency vs. CsA
P-gp	Caco-2	>50	1	>50× less
BCRP	MDCKII-BCRP	23	3	7.7× less
BSEP	Vesicles	1.42	0.42	3.4× less
MRP2	Vesicles	20.7	10	2.1× less
OATP1B1	HEK293	0.3	0.04	7.5× less
OATP1B3	HEK293	0.6	0.08	7.5× less
OAT1	HEK293	>50	>50	Same (no inhibition)
OCT2	HEK293	>50	>50	Same (no inhibition)
OAT3	HEK293	0.473	>10	>21× more
NTCP	HEK293	45.3	1	>45× less
MATE-1	HEK293	>100	>100	Same (no inhibition)
MATE-2	HEK293	>100	>100	Same (no inhibition)

BCRP, breast cancer resistance protein; HEK, human embryonic kidney; MATE, multidrug and toxin extrusion; MDCK, Madin–Darby canine kidney; OCT, organic cation transporter; P-gp, permeability-glycoprotein.

#### CRV431 Absorption and Distribution.

CRV431 blood and liver exposures following daily oral dosing were evaluated in several mouse and rat studies (Table 6). Across a large range of doses, 30–250 mg/kg per day, and dosing periods, 7–189 days, blood levels of CRV431 were consistently about 1–3 *µ*g/ml. This was also observed across a wide range of sampling periods from 3 to 24 hours postdose, suggesting a long half-life for CRV431. We also consistently observed much higher concentrations in livers, representing 5- to 15-fold higher levels than in the blood. High liver exposures may suggest pharmacokinetics of CRV431 favor hepatic delivery or targeting of CRV431. Mice from NASH and carbon tetrachloride models of disease had higher liver-to-blood ratios of CRV431 than normal rats.

**TABLE 6:** CRV431 concentrations in blood and liver Means ± S.D.

Species	Study Treatments	mg/kg per day	Day of Dosing	Hours Post-Dose	*n*	Blood *µ*g/ml	Liver *µ*g/g	Liver:Blood [CRV431] Ratio
Mouse	NASH	50	189	3	10	1.4 ± 1.5	11.6 ± 9.0	11.9 ± 8.5
Mouse	CCl_4_	50	42	4	9	1.2 ± 0.5	14.5 ± 2.9	15.4 ± 10.4
Rat	—	30	7	12	6	1.8 ± 0.3	11.7 ± 1.9	6.6 ± 1.1
Rat	—	30	7	24	6	1.3 ± 0.2	6.0 ± 1.4	4.8 ± 1.1
Rat	—	250	7	12	6	3.2 ± 0.7	23.4 ± 3.4	7.6 ± 1.8
Rat	—	250	7	24	6	2.9 ± 0.6	15.2 ± 4.0	5.3 ± 0.6

**Fig. 3.**
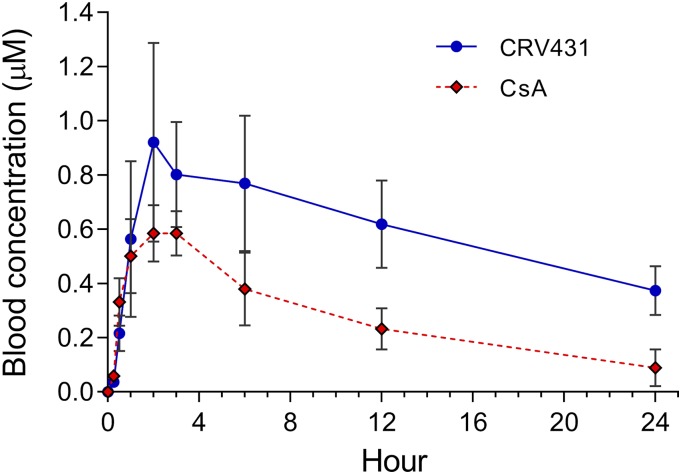
CRV431 pharmacokinetics in rats. Single doses of CRV431 or CsA at 10 mg/kg were administered by oral gavage to three male and three female adult Sprague–Dawley rats. Whole–blood concentrations of the two compounds were determined at the indicated time points. Data from male and female rats were combined because no statistical differences in drug exposures between genders were observed. Means and S.D. shown.

A more detailed pharmacokinetic analysis in normal rats following a single oral dose confirmed a relatively long CRV431 half-life that exceeded that of CsA (Fig 3). This was also exemplified by clearance values of 0.32 ml/min per kilogram for CRV431 and 1.11 for CsA, representing a 3.5-fold difference. CRV431 peak blood concentrations (Cmax) and AUC_0–24 hours_ exceeded those of CsA by a factor of 1.6 and 2.2, respectively, which may be due to differences in oral bioavailability and/or drug clearance.

#### CRV431 in the Carbon Tetrachloride Mouse Model of Liver Fibrosis.

**Fig. 4.**
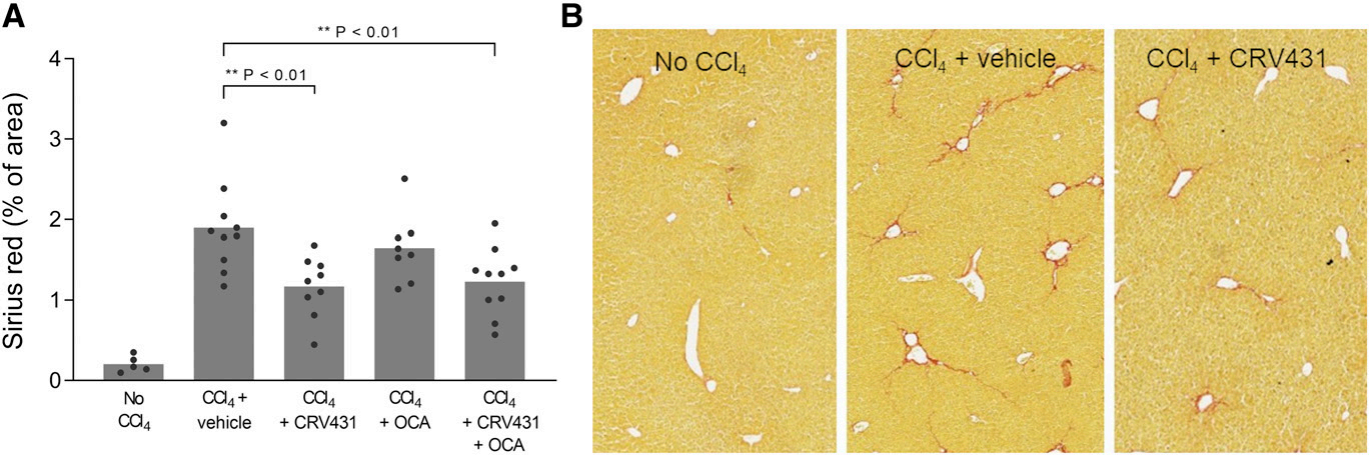
Liver fibrosis in the carbon tetrachloride mouse model. CCl_4_ was administered intraperitoneally to male C57BL/6 mice three times per week for 6 weeks. Vehicle or compounds were administered by daily oral gavage for the entire 6 weeks: CRV431 (50 mg/kg per day), obeticholic acid (10 mg/kg per day), or a combination of CRV431 and obeticholic acid (50 and 10 mg/kg per day, respectively). (A) Liver fibrosis was assessed at the end of treatment by measuring the relative area of sirius red staining in liver sections. *P* values from unpaired, parametric, two-sided *t* tests. (B) Representative staining of liver sections with sirius red.

The high levels of CRV431 in livers, inhibition of multiple cyclophilins, and other favorable properties described above suggested that CRV431 may be a good candidate for testing in liver disease models. One common model is chronic dosing of mice with the hepatotoxin, carbon tetrachloride (CCl_4_), which leads primarily to liver fibrosis. C57BL/6 male mice were dosed twice weekly with CCl_4_ for 6 weeks with daily oral dosing of vehicle or CRV431 (50 mg/kg per day). Obeticholic acid (OCA), a compound in development as a NASH treatment, was included as a comparator compound and dosed at 10 mg/kg per day CCl_4_-induced liver fibrosis, as demonstrated by 10-fold higher levels of collagen staining with sirius red in liver sections as compared with mice not administered CCl_4_ (Fig. 4). CRV431 treatment lowered the amount of sirius red staining by 43%, in contrast to OCA, which had no statistically significant effect. One group of CCl_4_ mice that were treated with a combination of CRV431 and OCA also showed fibrosis reduction to similar levels as CRV431 alone.

#### CRV431 in a Mouse NASH Model.

**Fig. 5.**
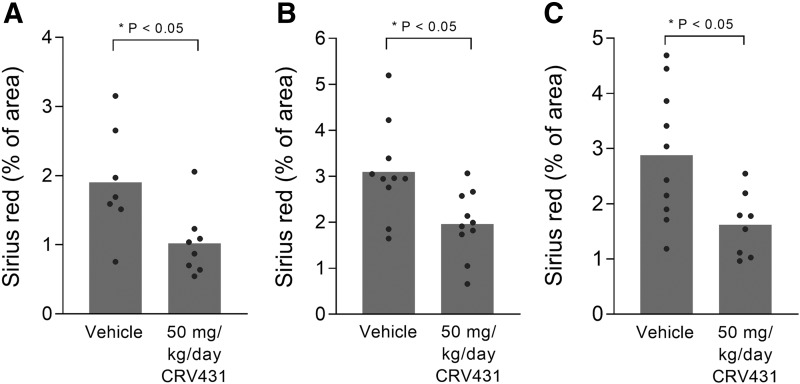
Liver fibrosis in mouse NASH model. Vehicle or CRV431 (50 mg/kg per day) was administered orally to NASH mice in three independent studies. (A) Week 8–14 treatments, (B) week 3–14 treatments, (C) week 20–30 treatments. Liver fibrosis was assessed at the end of treatment by measuring the relative area of sirius red staining in liver sections. *P* values from unpaired, parametric, two-sided *t* tests.

The second mouse model used to evaluate CRV431 efficacy was a NASH model in which C57BL/6 mice were given a single dose of streptozotocin at 2 days old and a high-fat diet starting at 3 weeks of age. Mice progressively developed obesity, liver steatosis, fibrosis, and eventually liver tumors starting beyond 14 weeks of age. Three separate studies were conducted in this model, varying in the start time and duration of CRV431 treatment. In similarity to the CCl_4_ study, we found that daily oral treatment with CRV431 at 50 mg/kg per day decreased the level of fibrosis in all three NASH model studies as determined by sirius red staining of liver sections (Fig. 5; Table 7). CRV431 was effective when administered either at early-to-intermediate stages of the model (i.e., week 3–14 and week 8–14 treatment), or at late stage of the model (i.e., week 20–30 treatment). Fibrosis levels were 37%–46% lower after CRV431 treatment compared with vehicle treatment. Statistically significant differences between vehicle and CRV431 treatment groups were also observed for body weight and NASH activity score (composite of steatosis, inflammation, and ballooning) in the week 3–14 treatment study. In the week 20–30 treatment study, fibrosis scores from the CRV431-treated mice were lower than for the vehicle-treated mice.

**TABLE 7:** NASH mouse model data Mean ± S.D.; *P* values from unpaired, two-sided *t* tests between vehicle and CRV431 treatment groups. Nonparametric *t* tests for scores and parametric *t* tests for all other variables. *N* values shown in Fig. 5.

	Week 3–14 Treatment		Week 8–14 Treatment		Week 20–30 Treatment	
	Vehicle	CRV431	Vehicle	CRV431	Vehicle	CRV431
Body weight	39.7 ± 6.0	33.5 ± 5.2 (*P* < 0.05)	30.0 ± 7.1	26.1 ± 5.1	48.9 ± 8.3	41.6 ± 8.9
Liver weight	1.60 ± 0.44	1.28 ± 0.28	1.28 ± 0.19	1.35 ± 0.28	4.17 ± 0.74	3.04 ± 1.19 (*P* < 0.05)
Blood glucose	333 ± 70	319 ± 79	467 ± 123	463 ± 143	442 ± 90	405 ± 134
Steatosis score	2.0 ± 1.1	1.3 ± 1.1	1.5 ± 0.9	2.0 ± 0.5	n/a	n/a
Inflammation score	2.3 ± 0.8	1.5 ± 0.7	1.8 ± 1.5	2.4 ± 0.7	n/a	n/a
Ballooning score	1.4 ± 0.5	0.9 ± 0.6	1.5 ± 0.8	1.8 ± 0.5	n/a	n/a
NAS score	5.7 ± 1.6	3.7 ± 2.2 (*P* < 0.05)	5.4 ± 2.4	6.1 ± 1.1	n/a	n/a
Sirius red %	3.10 ± 1.03	1.96 ± 0.72 (*P* < 0.05)	1.90 ± 0.79	1.02 ± 0.48 (*P* < 0.05)	2.88 ± 1.20	1.62 ± 0.57 (*P* < 0.05)
Tumor number	n/a	n/a	n/a	n/a	8.1 ± 2.8	4.5 ± 4.4
Tumor score	n/a	n/a	n/a	n/a	4.7 ± 1.9	2.25 ± 2.0 (*P* < 0.05)

n/a, not applicable; NAS, NASH activity score.

**Fig. 6.**
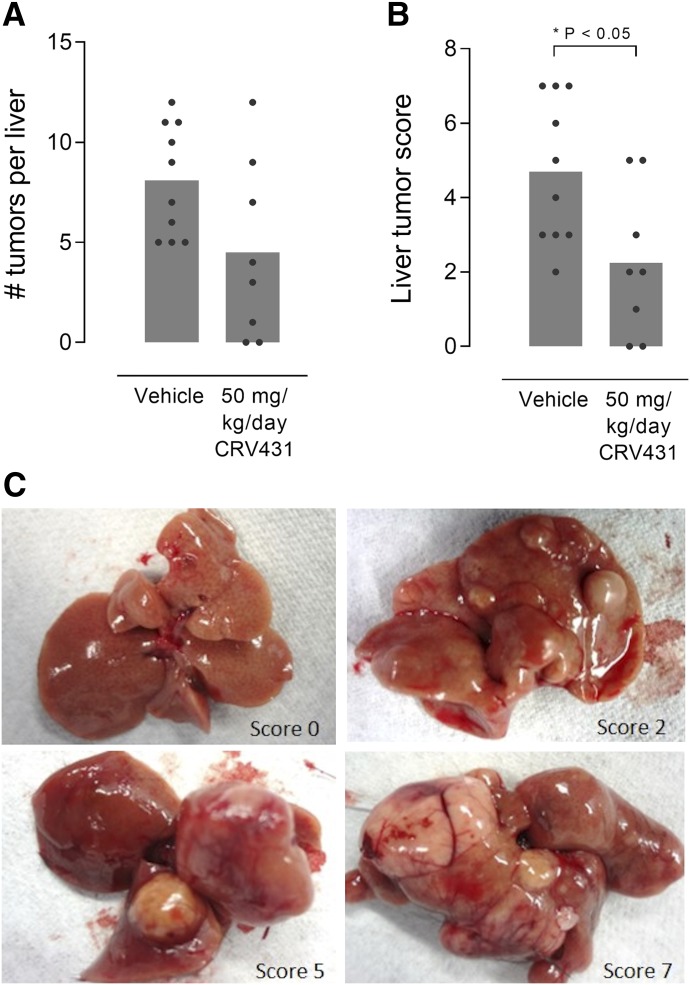
Liver tumors in mouse NASH model. Vehicle or CRV431 was administered orally to NASH mice from week 20 to 30 corresponding to a late disease stage characterized by liver tumor development. Tumor burden at the end of treatment was assessed by the number of tumors (A) and a composite score based on the number and size of tumors (B; 0–7 scale). *P* values from unpaired, nonparametric, two-sided *t* tests. (C) Representative images of liver tumors and tumor burden scores.

Mouse livers examined at week 14 had no tumors, whereas vehicle-treated mice at week 30 had an extensive tumor load, indicating that liver tumors developed sometime during weeks 14–30 (Fig. 6). Histopathological assessment indicated that the tumors were cellular in nature and therefore likely represented hepatocellular carcinoma. Moreover, in the vehicle control group all mice had liver tumors, and 3 of 10 livers had nodules that were 1 cm or larger in diameter. In contrast, CRV431 treatment from weeks 20 to 30 resulted in half the number of tumors, with smaller tumors on average, with 2 of 10 livers of CRV431-treated mice lacking tumors. A scoring system reflecting a combination of tumor number and size revealed that CRV431 decreased the tumor burden by 52%. Further studies are required to determine whether the reduced tumor load is mechanistically linked to the lower fibrosis levels or reflects a more direct effect on the cells.

## Discussion

Experimental and clinical evidence has accumulated over nearly three decades, indicating that antagonists of cyclophilins may offer clinical benefits. Many attempts have been made to develop cyclophilin inhibitors for clinical use, the most notable being alisporivir (Debio-025), which demonstrated good antiviral activity toward hepatitis C virus (Buti et al., 2015; Pawlotsky et al., 2015). However, all cyclophilin-inhibiting drug candidates eventually faced obstacles that halted their development. CsA was the original and still the only approved drug that inhibits cyclophilins, but its immunosuppressive activity limits its clinical use for indications other than transplantation and autoimmune diseases. Our testing showed that CRV431 has greatly diminished immunosuppression potency, high cyclophilin inhibition potency, and other properties that are favorable for clinical development for liver diseases.

CRV431 inhibited the isomerase activity of cyclophilins A, B, D, and G. However, CRV431 probably also blocks several other isoforms based on conservation of the active sites and by the observation that CsA binds to at least 11 of the 18 reported isoforms (Davis et al., 2010; Lavin and McGee, 2015). The pan-inhibitory activity is proposed to be advantageous because a single drug could simultaneously target multiple pathologic activities and minimize the risk, complications, and expense of combining multiple, single-pathway drugs. Anti-inflammatory, anticancer, and antiviral activities have been linked to Cyp A inhibition (Yurchenko et al., 2010; Tian-Tian et al., 2013; Lavin and McGee, 2015; Dawar et al., 2017b). Antifibrotic activities may be linked to Cyp B inhibition due to its role in collagen production (Choi et al., 2009; Cabral et al., 2014; Terajima et al., 2016; Gjaltema and Bank, 2017). Cytoprotective properties are linked to Cyp D inhibition and desensitization of mitochondrial permeability transition (Alam et al., 2015; Porter and Beutner, 2018; Springer et al., 2018). Targeting multiple pathways is likely a prerequisite for effectively treating chronic inflammatory and fibrotic diseases, and cyclophilin inhibition may be one way of achieving this goal.

Cyclophilins are abundant in eukaryotic cells and have important physiologic functions, so questioning their suitability as drug targets is reasonable. Genetic knockouts of individual isoforms A, B, and D in mice are generally well-tolerated, and in fact most studies document positive effects of cyclophilin knockout in disease models (Nigro et al., 2011; Elvers et al., 2012; Guo et al., 2016; Shum et al., 2016; Huang et al., 2017; Wang et al., 2018). More informative than genetic knockouts are the 35 years of clinical experience with CsA and its analogs. Although CsA can exhibit side effects such as nephrotoxicity, hypertension, and dyslipidemia, it can be taken lifelong by organ transplant patients under careful management. Moreover, many of the side effects appear to result from its immunosuppressive activity rather than cyclophilin binding. First, some of the clinical toxicities of CsA also occur with another calcineurin inhibitor, tacrolimus, which does not bind to cyclophilins. Second, the nonimmunosuppressive analog, alisporivir, was dosed to over 2000 patients at high doses and showed no renal toxicity or infections characteristic of CsA treatment and a moderation of other CsA-like side effects (Buti et al., 2015; Pawlotsky et al., 2015; Zeuzem et al., 2015; Stanciu et al., 2019). Finally, preclinical toxicology studies with CRV431 showed a remarkably good safety profile that has enabled clinical studies.

Several findings point to advantages of CRV431 over CsA and other previously described cyclophilin inhibitors. First, CRV431 is not expected to exhibit clinically significant immunosuppression, based on in vitro assays that showed at least 35-fold less immunosuppressive activity than CsA and based on our observations that CRV431 efficacy occurred in mice at peak blood levels of 1.2–1.4 *µ*g/ml, which is only slightly higher than the therapeutic range for CsA in transplantation—peak levels of 0.45–1.3 *µ*g/ml and trough levels of 0.1–0.35 *µ*g/ml (BC Transplant Provincial Health Services Authority, 2019). Second, the lower cytotoxicity potency of CRV431 means that there is less likelihood of side effects arising from excessive drug accumulation in organs. Third, CRV431 is predicted to have few drug–drug interactions or toxicities arising from membrane transporter inhibition, based on our in vitro profiling. Interestingly, CRV431 possesses similar physicochemical characteristics to those identified by Novartis as being critical for minimizing transporter inhibition by cyclosporins, which represent a significant improvement over alisporivir and CsA (Fu et al., 2014). Cyclosporine inhibition of several membrane transporters can affect the clinical disposition of other compounds, and CRV431 was noninhibitory or less potent than CsA toward all of them, with the exception of OAT3. The most notable transporter findings were the absence of permeability–glycoprotein inhibition in the Caco-2 transwell assay and the 45-fold lower activity toward the bile salt transporter, sodium-dependent taurocholic acid cotransporting polypeptide. A transporter of conjugated bilirubin, MRP2, also was slightly less affected by CRV431 than CsA. These latter findings suggest that CRV431 is less likely to impact bilirubin and bile salt transport, and results from preclinical toxicology studies are consistent with this prediction. Despite these positive in vitro and preclinical findings, it will be important to monitor patients for drug–drug interactions considering the effects that CsA are known to exhibit. One final advantage of CRV431 as a drug candidate for liver disease is that its steady-state accumulation in the liver, the intended site of action, was 5- to 15-fold higher than in the blood. This characteristic, however, is not unique to CRV431, as CsA also preferentially localizes to the liver (Wagner et al., 1987; Lensmeyer et al., 1991).

The decreased liver fibrosis observed in four studies with two mouse models is consistent with several previous studies that used CsA or other nonimmunosuppressive analogs. CsA and NIM811 administered to CCl_4_-treated rats lowered liver fibrosis, alanine aminotransferase, inflammation, transforming growth factor-*β*, tissue inhibitor of metalloproteinase-1, and other markers (Lie et al., 1991; Wang et al., 2011). In the rat bile duct ligation model, NIM811 did not reduce fibrosis but decreased liver necrosis and alanine aminotransferase (Rehman et al., 2008). In a clinical study of liver transplantation that compared CsA to tacrolimus, less post-transplant liver fibrosis was observed in steroid-free, CsA-treated patients (Levy et al., 2014). Antifibrotic effects have also been observed with NIM811 and MM284 in models of cardiac fibrosis (Seizer et al., 2012; Heinzmann et al., 2015). The physicochemical properties of MM284 dictate that it targets extracellular cyclophilins, which suggests that fibrosis is lowered at least partly by attenuating Cyp A-CD147–mediated inflammation (Iordanskaia et al., 2015). Similarly, in the unilateral ureter obstruction model, preservation of cell viability and lowering of inflammation by Cyp D knockout partly prevents renal fibrosis (Hou et al., 2018). Direct, antifibrotic effects on hepatic stellate cells, the primary cell type implicated in liver fibrosis, is another proposed mode of action. CsA, NIM811, and SCY-635 alter many activities in these cells in culture toward an antifibrotic phenotype, including changes in collagen production, matrix metalloproteinase and tissue inhibitor of metalloproteinase-1 levels, and transforming growth factor-*β* and mitogen-activated protein kinase signaling pathways (Nakamuta et al., 2005; Kohjima et al., 2007; Scorneaux et al., 2010). Future studies will attempt to identify the contributions of each cyclophilin isoform and their modes of action in liver fibrosis.

The significant reduction in tumor number and size by CRV431 in the NASH mouse model was supported by previous reports suggesting numerous possible mechanisms. The reduction in fibrosis may have limited tumor growth because hepatocellular carcinoma is regulated strongly by the extracellular milieu (Cox and Erler, 2014; Baglieri et al., 2019; Sircana et al., 2019). CRV431 may have restored levels or function of the tumor suppressor, p53, by blocking the binding of Cyp D to p53 or blocking p53 degradation through a Cyp A-mediated mechanism (Bigi et al., 2016; Lu et al., 2017). Cancer cells frequently express high levels of cyclophilins, which appears to support the elevated anabolic demands, cell proliferation, and hypoxia adaptations of the cells. For example, the transcription factor, hypoxia-inducible factor-1, binds to the Cyp A and B promoters and upregulates cyclophilin production (Kim et al., 2011; Zhang et al., 2014). A nuclear cyclophilin isoform, cyclophilin J, is upregulated in many hepatocellular carcinomas and facilitates cell cycle progression in part through cyclin D1 elevation (Chen et al., 2015). Extracellular Cyp A can increase cholangiocarcinoma cell proliferation through binding to CD147 receptor (Obchoei et al., 2015). Conversely, decreasing cyclophilins or blocking their function has been shown to suppress cancer cell proliferation and metastasis (Kim et al., 2011; Zhang et al., 2011, 2014; Choi et al., 2014, 2018; Brichkina et al., 2016; Guo et al., 2018). Thus, CRV431 may lower the risk of hepatocellular carcinoma not only indirectly by helping to normalize the liver parenchyma but also directly by suppressing cell proliferation or adaptive intracellular pathways required for carcinogenesis.

In conclusion, many investigators have identified the need and therapeutic opportunities of a potent cyclophilin inhibitor without the immunosuppression and other liabilities of CsA. Findings from the present study and previous studies endorse CRV431 as the strongest candidate to date to fulfill this role. Its ability to attenuate multiple pathologic and viral activities and its inherent disposition to the liver makes CRV431 particularly well suited for liver diseases of various etiologies. A single drug that could decrease both liver fibrosis and cancer incidence would be a valuable asset in the management of nonalcoholic, alcoholic, and viral hepatitis.

## Abbreviations

BSEP: bile salt export pump
CCl_4_: carbon tetrachloride
CFSE: carboxyfluorescein succinimidyl ester
CsA: cyclosporin A
Cyp A: cyclophilin A
Cyp B: cyclophilin B
Cyp D: cyclophilin D
HCC: hepatocellular carcinoma
IL-2: interleukin-2
Ki: inhibitory constant
MRP: multidrug resistance protein
NASH: nonalcoholic steatohepatitis
NFAT: nuclear factor of activated T cells
OAT: organic anion transporter
OCA: obeticholic acid
PBMC: peripheral blood mononuclear cell
PMA: phorbol myristate acetate
PPIA: peptidyl prolyl isomerase A
Vmax: maximum velocity
